# The Hospital. Nursing Section

**Published:** 1906-01-20

**Authors:** 


### The Hospital.

### Nursing Section.

Contributions for "The Hospital," should be addressed to the Editor, "The Hospital" Nursing Section, 28 & 29 Southampton Street, Strand, London, W.C.

No. 1,008.—Vol. XXXIX. SATURDAY, JANUARY 20, 1906.

### Notes on News from the Nursing World.

### THE RUSSIAN ADMIRAL AND THE NURSE.

Admiral Rodjestvensky has implicated a nurse in a statement which he has apparently been making for the purpose of trying to obtain credit for his exploded story about the Russian fleet being attacked at Dogger Bank by Japanese torpedo-boats. He affirms that while he was in hospital he came across a nurse who assured him that a Japanese officer, who was also there and was represented as being ill but was really wounded, had actually gone to England with torpedo-boats. The Admiral says that, having made "discreet inquiries," he subsequently ascertained that the officer's arrival in hospital, "coincided in a striking manner with the events of Hull, when account was taken of the time needed for his return voyage." If there was such a nurse as Admiral Rodjestvensky describes, and she betrayed, as he alleges, the secrets of the sick-bed, she merits condemnation. Whether in the hospital or in the private house, the confidences of patients to nurses should be scrupulously respected. In this instance, the patient, or the nurse, or the Admiral, has doubtless been the victim of a hallucination.

### A NURSE IN ATTENDANCE ON A CANDIDATE.

A remarkable incident of the General Election which is proceeding at the present time was the appearance of Sir Elliott Lees, who was a candidate for Birkenhead, at a public meeting attended by his medical man and his nurse. A short time ago he was kicked by a horse in the hunting field and was badly hurt. But he insisted upon personally taking part in the contest for the seat, and was conveyed from his carriage on a stretcher to the room in Queen's Hall where the meeting was held. There he was placed in a recumbent position upon a sofa on the platform and delivered a speech of an hour's duration, the nurse being on tenter-hooks throughout the whole of the time. But the patient, though he lost the election, does not seem to have suffered in health for his temerity.

### THE INSUBORDINATION AT GARTLOCH ASYLUM

At the monthly meeting of the Glasgow Parish Council and Lunacy Board last week a deputation from the Glasgow Trades Council attended in order to contend that the treatment of the five nurses who recently had been dismissed from Gartloch Mental Asylum was rather arbitrary, and to urge that they should be reinstated in order that they might have an opportunity of resigning. The Convener of the Gartloch Asylum Committee stated that the Committee entirely supported the action of the Medical Superintendent, and it being asserted that the latter had not the power of dismissing the nurses without appealing to the Committee, it was shown that by a standing order of the Council he was armed with authority to engage and dismiss every member of the staff. Apart from this, however, we think that in the interests of the patients the Medical Superintendent was bound to take the course he did, and the more so because the leaders of the insubordination had been in the Asylum for some years. They could not therefore even plead the poor excuse that, owing to inexperience, they did not realise the seriousness of advising a strike. It is to be hoped that the punishment which they have justly brought upon themselves by metaphorically presenting a pistol at the head of the Medical Superintendent will operate as a warning to any discontented nurses who imagine that the redress of grievances is most likely to be obtained by threats of revolt regardless of consequence to the sick and suffering.

### THE SUPREME ISSUE.

We think that the article entitled "A Plea for the Dying," which appears in another column, will be perused with interest and appreciation by many of our readers. It touches points of great delicacy as well as of great importance, and we may mention that our contributor, who is a male nurse, has told us, in confidence, the name of the patient to whom reference is made. He was a most eminent ecclesiastic, who had a wide circle of admirers in this and other countries. We are in accord with our contributor as to the need of his plea, and those who take up nursing as an avocation, and not merely as a profession, will not require to be persuaded of its validity. Patients who are obviously dying, for whom nothing more can clearly be done, so far as remedial treatment is concerned, should not be disturbed in order to have their temperature taken or their pulse felt, nor importuned to swallow medicines. In other words, nothing that is not absolutely essential should be attempted or said to prevent them from devoting the closing moments of their life to the religious exercises which then become the supreme issue.

### THE PROTEST OF THE IRISH NURSES' ASSOCIATION.

At the last meeting of the North Dublin Guardians a protest from the Irish Nurses' Association against the proposed scheme for training nurses in the North City Infirmary was read. The scheme is objected to by the Association on the ground that the period of training is not for three years; that a fee of £20 is asked; that on leaving the Infirmary certificated nurses of inferior training will be foisted on the public, to its danger and the discredit of the nursing profession; and that it is unfair to receive young women for training who are ignorant of the disadvantages from which they will suffer later on. The Association approved strongly of the proposal to abolish pauper nursing, and expressed their anxiety that the Guardians should start a scheme which would be equally beneficial to those who enter for training as well as to the sick poor. It was decided that a copy of the letter should be sent to every Guardian, notwithstanding the fact that a member of the Board observed that "they were not going to be lectured and bullied by every outsider calling itself an Association." We can understand that there may be practical difficulties in the way of complying entirely with the wishes of the Irish Nurses' Association, but the points submitted on their behalf merit the careful attention of the North Dublin Guardians, who have already shown their own desire to improve the standard of nursing.

### QUESTIONING CANDIDATES FOR APPOINTMENTS.

Within limits it is clearly permissible for Guardians to question candidates for appointments on their nursing staff. But an inquiry suggested by a Richmond Guardian savoured of impertinence. He desired to ask the candidates whether they were likely to get married shortly. The underlying motive for this proposal was the apprehension, not altogether unjustified by previous experience, lest there should again be vacancies at an early date. But at the instance of a lady member of the Board a change was made in the interrogation which rendered it innocuous. She thought that the candidates might be asked "if they would settle down if appointed." There is no objection to this, though, as we have no doubt that it would be answered in the affirmative as a matter of course, it seems superfluous. The possibility of getting married ought not in any way to be regarded as a disqualification for service as a nurse.

### MENTAL TRAINING FOR POOR-LAW NURSES.

At an inquest held this week at the Middlesex County Asylum evidence was given respecting the arrangements for the care of the sick at Willesden Poor-law Infirmary. It seems that a few days ago the wife of a coachman, suffering from broncho-pneumonia was received at this institution, and that on Tuesday last week when, as the nurse in attendance stated, she was in a dying condition, she was removed on a horsed ambulance to the Middlesex Asylum, a distance of ten miles, the journey occupying an hour and a half. She died two days later. Dr. Webster, Medical Officer at Willesden Infirmary, said that the removal of the patient was absolutely necessary, owing to the fact that they had no resident medical officer; and he thought that the transference to the Asylum was her only chance. He added that he had no nurse with whom they could properly entrust a patient like the deceased, although their nurses were thoroughly trained. The jury, while expressing the opinion that there should be a resident medical officer at the Workhouse, found that no blame attached to Dr. Webster or the nurse. We think that the jury have very properly exonerated the officials at Willesden; nor could a nurse who has not had mental training be expected to take charge of the case. But the incident shows the advantage of the staff of an important Poor-law infirmary including nurses who have enjoyed mental training.

### LECTURES TO QUEEN'S NURSES.

The report of the work of the Queen Victoria Nursing Institute at Reading for last year shows a very considerable development of the movement. The number of patients nursed was 556, as against 437 the previous year, and the number of visits paid 15,656, as against 12,474. An additional nurse was engaged in order to cope with the increased work. In spite of this extra expense, the treasurer's account of income and expenditure shows a balance in hand of nearly £50. The only unsatisfactory feature of the report is that the annual subscriptions were rather less than in the previous year, but, on the other hand, the income from donations, church collections, and donations from patients was £62 more. We are pleased to learn that the course of lectures on hygiene, given by Mrs. Clare Goslett, lecturer to the Queen's Nurses, realised upwards of £60, the substance of which has been placed to the credit of the capital account. This is a very encouraging result, and should have the effect of inducing committees in other towns to follow the example of Mrs. M. J. Sutton and her colleagues.

### THE QUESTION OF A CERTIFICATE

At the meeting of the Chichester Guardians last week the question of granting a certificate to a probationer who had left the Infirmary before her period of service had expired was discussed at some length. This was the probationer who had three days' leave to go to her father because he was ill, and did not return, of whom mention was made in our columns some weeks since. Dr. Barford strongly urged that the certificate should be granted, and though the Chairman objected on the ground that the probationer had not treated the Guardians with adequate respect, only two members of the Board voted against the proposal. We agree with the majority that in the circumstances it would have been very hard on the probationer if she had not been given her certificate, with the endorsement, as suggested by Dr. Barford, that she left of her own accord to nurse her father.

### CHARGES AGAINST NURSES.

From time to time unfounded charges of cruelty are made against nurses, especially those engaged in mental asylums. A striking instance occurred recently in the case of a nurse at a county asylum. She was on visiting-room duty, and a newcomer was asking people, who had been visitors for some time, as to the treatment of patients. "Oh," was the reply, "they treats the poor souls cruel here; why, they gives 'em a bath every week."

### THE LADY AMPTHILL NURSES' INSTITUTE.

We are informed that the Lady Ampthill Nurses' Institute, to which reference was made in a recent issue, has at present a staff consisting of a matron, superintendent, and ten nurses. The salary for English trained nurses is £5 a month, with board and uniform. A month's leave in the year is allowed. The Institute cannot undertake the responsibility and expense of arranging to take nurses out, but will give appointments in India to suitable applicants. Each nurse is engaged for three months on probation, and if, during that time, she discharges her duties satisfactorily, the lady president and committee give her a position on the staff. Any other details can be obtained from Miss Tew, Matron of the Institute, Harrington Road, Chetput, Madras, India.

### THE ADA LEWIS NURSES.

Although, in consequence of Mrs. Lewis-Hill's illness, there has never been any formal opening of the Ada Lewis Nurses' Institute, the work is going on steadily and the progress has, in fact, been so considerable that it has been decided to engage a fourth nurse. Since the Institute was opened, between October 28 and December 22, there were thirty-three cases, to which between eight and nine hundred visits were paid. A large number of these have been operation cases, including one of a woman who was run down by a motor-car and very seriously injured. The cases, of course, vary greatly, and some are light, while others involve a great deal of attention. The working-hours of the nurses are from 8.30 a.m. to 1 or 2 p.m., and again from 4.30 p.m. to 8.30 p.m., with two hours off duty in the middle of the day; but hard and fast rules as to hours and meals are not practicable. The fees charged by the Institute are not employed in the reduction of establishment expenses, but will accumulate for the provision of similar homes in other districts.

### THE NEW NIGHT SUPERINTENDENT OF NEWCASTLE INFIRMARY.

It is interesting to learn that in response to the advertisement which appeared in The Hospital last month there were thirty-six applications for the post of night superintendent at the Royal Infirmary, Newcastle-on-Tyne, and that some of the candidates possessed certificates of efficiency granted by important hospitals. The selected candidate is Miss Rigbey, who was trained at St. Bartholomew's Hospital, and has enjoyed the advantage of experience in connection with the housekeeping department at the Nurses' Home. She has also been sister at the Samaritan Free Hospital, and her record of thorough training in the details of nursing work quite justifies the choice made.

### PROMOTION OF A LIVERPOOL SISTER.

The newly-appointed Matron of the Noble Hospital, Douglas, Isle of Man, is Miss Esther Bridson, of the Royal Southern Hospital, Liverpool. She was trained at the Royal Southern Hospital, Liverpool, and for the last nine years has been sister of some of its most important wards, fulfilling her various duties not only to the satisfaction, but to the admiration of the entire staff. While the staff of the Royal Southern Hospital are losing a valuable sister, the Noble Hospital is, we believe, gaining not only an efficient Matron, but an industrious worker, whose quiet example and unflagging interest will always be exerted to promote that peace and harmony, without which the most strenuous and energetic of workers can bring no true comfort to the hospital for which they are working. In returning to her native place, Miss Bridson will take with her both regret for her loss and the heartiest desires for her welfare from all her fellow-workers at the Royal Southern Hospital.

### WEDDING OF A SUPERINTENDENT NURSE.

An interesting wedding took place on Wednesday afternoon last week at Sandbach Heath Church, Cheshire. The bride was Miss Clara Cattrall, superintendent nurse at Sandbach Union Hospital, and the bridegroom, Mr. Harry Walker, of Holmes Chapel. The bridesmaid was Miss S. Cattrall, superintendent nurse of Chorley Union Hospital, sister of the bride. Both wore their indoor uniform. The presents were numerous and handsome.

### DEATH OF A DISTRICT NURSE FROM TYPHOID FEVER.

It is not often that we have to record the death of a district nurse from typhoid fever. But we regret to learn that last month Miss Frances Birkhead, who was working in a very insanitary area in Newton-le-Willows, contracted the disease and succumbed to it. She was most devoted in the pursuit of her avocation, and was greatly esteemed among the poor in whose midst she laboured. Miss Birkhead was a member of the Royal National Pension Fund.

### VICTORIAN TRAINED NURSES' ASSOCIATION.

The Conjoint Board of Examiners to the Royal Victorian Trained Nurses' Association held the summer examinations on December 6 and 7. Sixty-seven candidates presented themselves for their final; forty-four in Melbourne, three in Beechnorth, ten in Ballarat, and nine in Bendigo.

### PRIZES FOR THE FINEST BABIES.

A novel feature of the annual festivities at the Acton Nursing Institute, when upwards of seventy old maternity patients who had been attended by nurses from the Institute during the last twelve months assembled, consisted of prize competitions for the finest babies under three months old. Three prizes having been duly awarded, the mother of a fourth baby almost equal to the prize-winners was given a consolation prize, which she greatly appreciated. The festivities, commencing at an early hour of the afternoon, were continued throughout the evening, concluding with a selection of up-to-date records on the phonograph, songs, comical sketches, and a ghost story.

### SHORT ITEMS.

Miss Ethel Williams, the new night superintendent at St. Luke's Hospital, Halifax, has been sister in that institution, and her appointment is therefore a promotion.—Miss A. K. Travers, who was trained at the Sussex County Hospital, Brighton, and who for five years has held the post of assistant matron in the same Institution, has undertaken the working of Mrs. Butcher's Surgical Home, 7 and 8 Osnaburgh Terrace, Portland Place, London. During her twelve years at the Sussex County Hospital Miss Travers also held the post of theatre sister, ward sister, and night superintendent.

### The Nursing Outlook.

"From magnanimity, all fear above;
From nobler recompense, above applause,
Which owes to man's short outlook all its charm."

### THE INSPECTOR AND THE POOR-LAW NURSE.

OUR columns are constantly bearing witness to the fact that the conditions of nursing in Poor-law infirmaries are steadily improving. At times there is even room for the doubt whether there is not a disposition to extravagance in one direction. When we hear of nurses' homes built and furnished at the cost of the ratepayers, more luxurious than those which are attached to great general hospitals supported by voluntary contributions, we begin to wonder where the line will be drawn. There is always a danger lest a period of unnecessary expenditure should be succeeded by one of blighting parsimony, and therefore we earnestly hope that sympathetic guardians, in their laudable desire to make the institutions under their control as attractive as possible to nurses, will not expose themselves to attack on the ground that they are lax in their use of public money. Up to a certain point we are convinced as to their ability to carry the ratepayers with them. The overwhelming majority of the latter, we are sure, wish that the nurses who perform the arduous duty of tending the sick in the wards of Poor-law infirmaries should be comfortably housed, should not be overworked, and should not suffer in health from hide-bound regulations in respect to diet. These being the vital issues to Poor-law nurses, it would be a matter for the utmost regret if, in consequence of protests arising from apparently unnecessary expenditure, there were any general disposition to put back the hands of progress.

Meanwhile, we do not think that the efforts of the inspectors of the Local Government Board to elevate the standard of Poor-law nursing and to increase the number of fully qualified nurses, are always as much appreciated as they deserve to be. In former days it is true that only scanty space was allotted in their annual reports to the nursing, and even to-day we do not consider that it has the prominence it merits. But there has been a marked change in recent years, and it is simply doing the inspectors justice to acknowledge the services which they have rendered, whether by deploring the employment of pauper attendants in the sick wards, the engagement of untrained persons as nurses, the insufficient provision for the accommodation of the nursing staff, or the mistake of not vesting sufficient authority in the superintendent or trained matron. The measure of success which has attended their attempts to stimulate action by the Department, as well as to influence the minds of the more cultivated and impressionable guardians, will, we trust, encourage them to continue to lose no opportunity of furthering what still remains to be done before it is possible for the advocates of reform in Poor-law nursing to be contented with the situation.

The inspectors have not only eyes to see—they have chances during their visits to the workhouse infirmaries of learning the weak spots in the system, and we have a right to require that they will make the most of them. The State pays them salaries primarily in order to ensure the proper care of people who cannot from various causes take care of themselves. But it is also expected, in addition, that they will not overlook the welfare of those who, like themselves, are public servants, but who cannot depend upon guardians, torn by divided opinions, to always understand their claims to consideration. We are glad to observe that one of the inspectors, in his report for last year, shows that he has not closed either his eyes or his ears when he has been on his rounds. He has seen, for example, that the fixed weekly diet which so usually prevails is open to many objections; and he is persuaded that it would be practicable to make an arrangement whereby the nurses might be fed well and pleasantly. There ought, of course, to be a greater variety of food, and, as we agree with Mr. Baldwyn Fleming that it is obviously to everybody's gain that the result he desires should be obtained, steps should be taken to bring it about.

Mr. Fleming has also noticed that the probationers and nurses feel that the Superannuation Act is defective. As it is open to guardians to determine their employment at any time without consulting the Local Government Board, there is obviously no rule to prevent them from being dismissed in order that they may be deprived of superannuation which is almost, but not quite, due to them, and we are not surprised at the existence of dissatisfaction. There are also still many smaller institutions in which the sleeping accommodation is notoriously defective. As to overwork, we published only last week an authentic statement that in one London Poor-law infirmary there is a single night nurse for 90 patients, whereas in others the night nurse has only 30 under her charge. It is open for inspectors to ascertain how far these and other disparities prevail, and to lay stress upon them. The more they make it clear that nothing escapes the range of their vision, that nothing will hinder them from embodying in their reports anything they see in need of alteration, and that nothing will deter them from protesting when their recommendations are ignored, the more surely will they be recognised as promoters of the best interests of Poor-law nurses—interests which must of necessity be identical with those of the community.

### Abdominal Surgery.

By HAROLD BURROWS, M.B., F.R.C.S., Assistant Surgeon to the Seamen's Hospital, Greenwich, and to the Bolingbroke Hospital, Wandsworth Common.

### TUBERCULOUS PERITONITIS.

The peritoneum sometimes becomes affected with tuberculosis as part of a general infection, obvious manifestations of that disease being present in other parts of the body—the lungs, meninges, joints, lymphatic glands. In such cases the abdominal lesion probably does not supply the chief characteristics of the patient's illness, appearing merely as a subsidiary and perhaps late complication of a grave widespread malady.

In other instances tuberculous peritonitis occurs in the absence of any distinct or severe affection of other organs of the body, and it is mainly to these cases that the following remarks are directed.

### SYMPTOMS.

The disease is most commonly seen in children, and usually it is difficult to recognise in the early stages. Occasionally it commences as an acute illness, with sudden and severe abdominal pain and rapid enlargement of the abdomen, due to the effusion of fluid. More often the onset is gradual, and of the kind which text-books describe as "insidious." The patient, who perhaps belongs to a tuberculous family, and is a delicate-looking child, at first is noticed to be peevish and fretful. His appetite becomes capricious, he begins to waste, and grows languid and pale. Presently he complains of pain in the abdomen, which is accompanied by diarrhœa or constipation, or these may alternate. The temperature chart, if one be kept in this stage, shows slight pyrexia.

Up to this point there may be no definite evidence of the true nature of the child's indisposition; indeed, he may improve in health after a while, and gradually get quite well again without any suspicion having arisen as to the presence of abdominal tuberculosis. More often he grows worse, and definite symptoms arise. One of the chief of these is swelling of the abdomen, and this may be the first event to alarm the mother, and to cause her to seek medical advice for the child. The swelling is due to the accumulation of clear yellow fluid in the peritoneal cavity. This condition is known as ascites. Sometimes the fluid accumulates in such large quantities as to cause great distension of the abdomen, urgently requiring relief. More frequently the distension is not excessive, and does not itself call for active surgical measures.

The other definite symptom which may result from peritoneal tuberculosis is intestinal obstruction. This happens at a later stage than the ascites, and is due either to the matting together of neighbouring coils of intestine so that they cannot perform the movements necessary to propel their contents onwards, or to actual constriction of a portion of the bowel by scar tissue which has resulted from the inflammatory process in the peritoneum.

### TREATMENT.

In the early stages much benefit may be derived from what is now generally known as the open-air or hygienic treatment. This should be carried out in the country, as the foul atmosphere of a large town is unsuitable for any sick person, and especially for one who is suffering from tuberculosis. Abundance of fresh air, at night as well as at day; sunshine; good, nourishing food, including especially cream, raw meat juice, and eggs—these are the best medicines, and, properly used, they will cure most cases if applied early enough, and with sufficient skill and determination. Sometimes operative treatment is adopted. In the first place, opening the abdominal cavity and washing it out with normal saline solution has been attended with a certain amount of success as a remedial measure. In the second place, operation may be required for the relief of intestinal obstruction, which, as already stated, may be produced in the later stages of tuberculous peritonitis.

The nurse in charge of a case of tuberculous peritonitis should be especially careful to record every day the condition of the bowels and also the girth of the abdomen. The latter serves as a rough measure of the amount of ascites present, though it also caries with the amount of intestinal distension. The girth is taken by passing a tape measure round the body at the level of the umbilicus. When possible, the patient should be weighed once a week and his weight recorded. It is hardly necessary to point out that an increase of weight may be discounted by an increase of the ascites, but a real gain of weight due to improved nutrition is a valuable sign of amendment

### ASCITES.

The effusion of fluid into the abdominal cavity in cases of tuberculosis usually is not excessive, and does not require active measures for its relief. But there are other conditions—cirrhosis of the liver, cancer of the peritoneum, and some cases of cardiac and renal disease—which frequently cause very large amounts of fluid to collect, so that the abdomen becomes greatly distended, the movements of respiration hampered, and the action of the heart embarrassed; and it is necessary to give relief by drawing off the excess of fluid. This is done by the operation known as "tapping." The apparatus required for this are a Southey's trocar and cannula, or a fine aspirating trocar and cannula, with a pilot in case the cannula becomes blocked; some india-rubber tubing which fits the cannula, and is for the purpose of conducting the ascitic fluid into a receptacle placed on the floor. The patient should be seated in a comfortable armchair or propped up into a sitting posture in bed. This position is to allow the fluid to collect in the lower part of the abdomen, the intestines floating to the upper part, where they are out of the way of injury. It is essential that the patient empties his bladder immediately before the operation, as if distended it is liable to be wounded by the trocar. Some stimulant—brandy, ether, or strychnine—should be at hand in case the patient becomes faint, as sometimes happens when the tapping is in progress. The lower part of the abdomen is prepared beforehand as for operation, and the instruments are boiled. A test-tube serves well enough for boiling the trocar, cannula, and pilot, but the trocar must not be placed in the test-tube point downwards, or it will be blunted. The surgeon sits facing the patient, and a little to his right side. The trocar and cannula, having been thrust through the abdominal wall, the trocar is withdrawn, and the india-rubber tubing is attached to the cannula so as to convey the ascitic fluid into the receptacle prepared for it. Some sterile gauze is placed round the cannula, and kept in position by a small piece of strapping. Care must be taken to prevent the weight of the india-rubber tubing from dragging upon the cannula, or else much pain and discomfort may be caused.

A watch is kept for any signs of syncope. If these arise some stimulant must be given, and the escape of ascitic fluid stopped by pinching the india-rubber tube until the patient has recovered. Faintness is more likely to ensue in the course of a first tapping, especially if a large trocar has been used.

As it will take some hours perhaps to remove a large collection of fluid, it will probably be left to the nurse to observe and report when the fluid has ceased to escape. Occasionally it may be entrusted to her to remove the cannula. To do this the cannula is smoothly and firmly withdrawn. There may be a little bleeding from the site of the puncture, but pressure with a piece of gauze applied with a finger to the bleeding spot will soon stop the hæmorrhage. A piece of gauze is then applied and fastened in position with collodion or strapping.

The quantity of fluid withdrawn should always be measured, and the amount noted on the chart.

### The Nurses' Clinic.

### THE NURSING OF CÆSARIAN SECTION IN A GENERAL HOSPITAL.

Cæsarian Section is the operation of opening into the uterus of a pregnant woman through an abdominal incision to extract the fœtus. The reason to make such an operation necessary would probably be a contracted pelvis following on early rickets, infantile paralysis, or some other such childish deformity which has obstructed the growth, leaving a malformation.

The bony ring which forms the pelvis, through which the child must pass in order to be born, being contracted, will not allow of the passage of the head (which is the largest and hardest part of the unborn infant, and which makes way for the other parts of the body). The process of natural labour being thus obstructed, the surgeon finds it necessary to remove the child by artificial means; he may do this in two ways, either by the operation of *Craniotomy* (crushing the skull of the fœtus before delivery), or *Cæsarian section*; his object in the latter case being to give the child as well as the mother a chance of life. Cæsarian section is oftener done in a maternity hospital than in a general, so that it is quite possible for a nurse to have a full general training without having an opportunity of nursing a case of this kind.

When a case for Cæsarian section is admitted to a general hospital it usually means immediate operation. The patient will probably have been in labour for several hours.

The nurse's first duty is to get the patient to bed with hot-water bottles; a bed-bath may be given if time and the condition of the patient will allow of it.

The same preparation of the skin will be necessary as in all cases for abdominal surgery, after which a compress of lint, wrung out of some antiseptic solution, may be applied. An enema and vaginal douche may be given if ordered by the doctor. The bladder must be emptied by a catheter before operation, but the parts being so much swollen, should the patient have been long in labour, it is a very difficult matter to pass a catheter except when the patient may be held in the lithotomy position, which can be easier done on the theatre table than when the patient is in bed.

The principal instruments required for the operation will be scalpel, bistoury, retractors, vulsellum forceps, artery forceps, dissecting forceps, long clamp forceps, scissors, needles (curved and straight), and needle holding forceps.

Fine silk will be required for stitching up the uterine incision after the fœtus has been extracted, stout silk for tying the umbilical cord, and some silkworm gut sutures for closing the abdomen. An irrigator filled with some antiseptic solution and douche nozzle ought to be in readiness in case the surgeon wishes to give the patient a vaginal douche, and a catheter. The theatre sister must take care to have a good supply of sterilised towels, which will be used by the surgeon to surround the uterus after the abdomen has been opened; and a blanket to receive the child.

When the surgeon has finished his operation he usually fixes a tight binder over the ordinary bandage and dressings. After operation the patient is got to bed in the ordinary way with hot-water bottles, a pillow under her knees, and an abdominal cradle to lessen the weight of the bedclothes. For the first forty-eight hours the patient will probably suffer from shock; if so the bed may be raised at the foot with blocks, stimulant and saline enemata may be given if ordered. The warmth of the body must be kept up with hot-water bottles and blankets.

The nurse must place her hand firmly on the patient's abdomen each time sickness or retching occur, in order to save rupture through the wound or bursting of the stitches.

Nothing ought to be given by mouth for several hours. When the sickness has passed off sips of hot water may be given at intervals, afterwards substituted with milk and kali, beef-tea, chicken-soup, etc., for the first few days and until the bowels have acted. Light nourishing diet may then be given.

The patient, being also a maternity case, will have a vaginal discharge called the "lochia." This discharge for the first few days should be of a bright red colour, similar to the menstrual flow, and fairly profuse. It should gradually become paler in colour and decrease in quantity, until from the tenth to the twenty-first day it should cease altogether. The pads will require frequent changing, especially during the first few days; the nurse must have her hands surgically clean each time she changes the pads, in case of infecting the patient with any septic matter, and she must also carefully sponge all round about the parts with some weak antiseptic solution.

If the discharge should become at all dark in colour or offensive in odour the doctor ought to be informed without delay, and vaginal douching may be ordered.

**THE NURSES' CLINIC**—*Continued.*

If the mother is fairly strong and the doctor wishes her to nurse the child, the infant should be put to the breast, but not for several hours after the operation.

After each nursing the nipples must be washed with warm water and kept very clean.

The milk comes slowly at first, but after a few days it will increase.

The patient may be considered in too weak a condition to nurse; in that case the breasts, if causing pain, may require to be exhausted for a few times, and belladonna applied as plaster or ointment, if ordered, until the milk has ceased to come.

A four-hourly temperature chart must be kept during the first week. There may be a slight rise of temperature about the third day, but if no complications have arisen, this will not be serious, and the temperature will soon return to normal.

The urine ought to be measured for the first week, a record being kept, in case of any suppression. The catheter may be passed, if necessary, at intervals of eight hours, but the patient ought to be encouraged to pass urine herself from the first.

An aperient is usually given on the third night after operation. After this the bowels must be kept regular, aperients being given when necessary.

The patient ought to be sponged down with warm water and soap daily, her back being carefully washed, rubbed with methylated spirit and powdered with starch powder. at least twice during the twenty-four hours. The abdominal binder will require to be tightened from time to time as the uterus contracts, care being taken not to disturb the dressings.

The surgeon will probably remove the sutures about the tenth day. The wound ought then to be healed, and only requires rest to strengthen the union.

The patient must lie quiet for a few days longer, then may sit up in bed, and probably about the end of three weeks from the operation will be allowed to get up, if everything has gone on satisfactorily.

The chief complication to be feared for the first few days after this operation is "puerperal fever."

The symptoms would be rigors, a rise of temperature, quick pulse, pain and tenderness in the abdomen, dry tongue, vomiting, and sometimes diarrhœa. The discharge from the vagina will be offensive or perhaps suppressed. This fever is nearly always fatal. About the end of the second week another complication may take place—phlegmasia alba dolens ("white leg"). The symptoms would be pain and swelling of the leg, usually the left, and a slight rise of temperature for a few days.

The principal treatment is rest and elevation of the limb, which ought to be wrapt in cotton-wool, a cradle being used to lighten the weight of the bedclothes. The leg ought to be disturbed as little as possible, and never rubbed until the swelling and pain have completely gone, which generally takes from three to six weeks.

*Management of the Infant.*—The child being taken from the uterus by the surgeon, the umbilical cord tied and cut, is received in a blanket by a nurse.

The infant will probably be in an asphyxiated condition, and will require to be revived by artificial respiration, slapping about the thighs, back, and chest with cold and hot water alternately, etc., to stimulate circulation. When the child cries and shows signs of life he may be taken near a fire for warmth. His body must be rubbed all over with some simple oil to soften the sticky whitish material with which it will be covered; the eyes, nose, ears, and mouth must be wiped and thoroughly cleansed from foreign matter. A warm bath may then be given, after which the baby ought to be dried and powdered with starch powder all over the body, especially in the groin, axilla, and any parts where folds of skin come in contact. The umbilical cord after being carefully dried, should be dusted with starch and boric powder and wrapped in a pad of lint or cotton-wool and fixed with a flannel binder, which ought to be sewn, not pinned. The cord should be disturbed as little as possible and will fall off about the fifth day; a pad must be kept firmly bound on the umbilicus for some weeks to save hernia. The child ought to be examined carefully by the nurse for any abnormalities of birth—harelip, cleft palate, imperforate anus or urethra, marks or bruises about the head or other parts of the body. The infant may then be dressed and put to bed with hot-water bottles, well wrapped up to keep warm. He will not require to be fed for several hours.

### Incidents in a Nurse's Life.

### A DOCTOR'S BRAVERY.

It was in the East End of London on a sweltering hot day at the end of June. The house surgeon of a well-known hospital was passing down a filthy back street when he was startled by a loud banging at one of the top windows of a house. On looking up he caught sight of a man's white face which even at that distance looked alarmingly drawn and haggard. Seeing the doctor looking up, the man beckoned frantically and pointed at his open mouth. It took only a few minutes for the doctor to run up the stairs of what seemed to him an empty house, and push open the top-room door. There he found the man gasping for breath, still pointing to his mouth and clutching his throat. In a very short time the doctor grasped the situation; the man was suffering from diphtheria which was then rampant in the district, and the people in the house, having probably been warned of the infectious nature of the disease, had fled from the house leaving the man to die alone. The doctor at once realised that if the man were not relieved almost immediately he must be suffocated, so he pulled the table—almost the only piece of furniture in the dark stuffy room—to the window, made the man lie down upon it and drawing him to the far edge, opened his penknife and performed tracheotomy. The diphtheritic membrane was at once coughed up through the opening made and the man breathed again fairly freely, the doctor meanwhile keeping his finger in the opening made. It is almost impossible to think of worse conditions under which an operation could be performed—no antiseptic precautions, no sterilised swabs, no tracheotomy tube, nothing to wipe off the blood and poisonous membrane but the doctor's own handkerchief, and only a pocket penknife, by no means a new one, to do duty for a scalpel! The operation over, the next thing was to get help. The doctor scribbled on a piece of paper torn from his pocket-book with his one disengaged hand, a note to the surgery sister: "Please send all things necessary for nursing patient who has had tracheotomy performed, also a tracheotomy tube, etc., with a nurse who can stay all night with a patient. At once, please, to top story, No. 10 —— Street." This precious note he had to await his chance to despatch. After a few minutes of anxious watching he espied an urchin who seemed a likely messenger. Having attracted his attention by shouting, he first of all threw down the note, asking the boy to take it as soon as possible to the address of the hospital, emphasising his injunctions to be as sharp as he could with a sixpence. Fortunately the boy proved trustworthy, and the sister was not long in despatching what the doctor required, together with the nurse.

With the help of the doctor and by procuring a few necessities, the patient was made fairly comfortable for the night, though nothing, unfortunately, could be done to get rid of the dirt and vermin with which the room was infested. Very early in the morning, to the inexpressible relief of the nurse, who was, needless to say, exceedingly anxious about her patient in such insanitary surroundings, the man was removed in an ambulance to the hospital, and, wonderful to relate, in due course of time made an excellent recovery. Had it lain in that nurse's power to apportion awards, the doctor who performed this deed would have found himself the surprised recipient of the Victoria Cross—"For Valour."

### A Plea for the Dying.

BY A MALE NURSE.

So great an advance has been made during the last decade in the realm of nursing that it would seem at first as if there could be no room for any further improvement. And in the broadest sense this is most certainly true - in our own country at least—and all due honour be to those who, in the face of countless difficulties, do battle by the side of the sick and the sad. So sensible am I of this fact that it is with a feeling of profound humility that I venture to touch on this subject, so near to my own heart. Yet I trust I may not be misunderstood, and that no one will take as a rebuke what is only intended as a gentle hint from one who, like themselves, has often kept watch by the bedside of the dying.

In all well-managed hospitals it is certainly true that the care of the dying is the most important duty of the whole ward work. It is put into the hands of the most highly skilled members of the staff, and is generally entrusted to the care of the sister or the nurse in charge. The resident medical officer is called to the bedside at once if there be any sudden or unlooked-for change; he pays frequent visits even in the most normal cases, and in only rare instances is he not present at the moment of death. This may not be always so where the patient is being tended in his own home, for a larger share of responsibility is then often expected from the nurse; but I venture to think there can never be any real lack of care under these or any other circumstances where a duly qualified person is in charge.

But is not this duty sometimes overdone? Have not the truly well-meant labours at times a tendency to become wearisome and a burden, even though the one desire of the zealous nurse is to be of the greatest comfort and use—to stay, if possible, the hand of Death? Upon the sick man has come a change—a change for the worse. There can be no mistaking its dread importance, for, having once seen the like, its symptoms cannot be misread. It is the nearing of the "last agony." It envelops him and stamps upon him an expression which is not of this earth, and we who watch by his side know in our hearts that this is surely the end. The bitterness of all pain is over; the patient is being gently borne on towards the great goal. Then, although the laboured respiration and the quickened, running pulse tells more convincingly than aught else that the sands of life are fast running down, with almost feverish haste the patient is aroused, perhaps, first of all, with the application of sundry hot-water bottles, or, it may be, by the clinical thermometer being thrust upon him, or the insertion of the hypodermic needle.

Next, as quickly as human agency can devise, brandy and other restoratives are brought, or some highly concentrated food or another, and every available effort is made to further the consumption of these products, all equally good and invaluable in their way, but, alas! sadly the reverse just now.

To me it seems to be almost certain that this brief hour of the agony, the very thought of which has often been fraught with dread and terror in our minds, is in the end shorn of all these, except, possibly, in a few—a very few, isolated cases. Instead upon the world-worn pilgrim there comes a soft, restful calm as the memory of an anxious past slips gently from him. The entire freedom from the pain which has been pursuing him for days and weeks, nay, perhaps for months past, is delicious in the extreme. The sweet, gentle peace now upon him brings with it a sense of almost perfect comfort.

It is at this time—(I am speaking from personal observation)—that he may experience a marked desire for spiritua consolation. It is possible that he may have expected death for some time. He has longed to make his peace with God and with all men, and at this time he realises that he is better able to see to this than at any other period during his illness. His thoughts gently turn to higher things, his spirit hovers heavenwards. The after-glow of eventide sheds its departing rays on the erstwhile troubled mind and body with a quiet warmth of tender peace which is wholly satisfying. Perhaps some stray thought may cross his mind that this is the end of his journey, but he shows no sign of alarm.

It is all so perfect, this soft touch of the Great Father's hand. It is sad to think this well-earned repose is sometimes broken by the miscalculated zeal of the nurse. Remember, I speak only of that last phase, the "agony" of death, the hour in which no doubt can exist as to the future development of the disease.

It may be, it frequently is, acutely painful for us who watch these symptoms; the laboured respiration, the seeming struggle and the very helplessness of the patient, all appeal to our most intense sympathies, but this should not tempt us to do what is obviously impossible. The attempt to force stimulants or food upon him now is cruel in the extreme, for even if the action of swallowing should not be particularly difficult, it serves to arouse him, and cannot be of any real use. Indeed it often proves the reverse, for the intellect becomes dulled, or else the brain excited, and he becomes distressingly restless.

But some nurses may say—not without reason—"We are sent to minister to the diseased body, we have nothing to do with the patient's soul; we leave spiritual matters to the clergy." This, to-day, is true, but I rejoice that it is just as much our duty also to see that every obstacle is removed from their path.

There is no heed in this last hour of life to keep taking temperatures or feeling for the almost imperceptible heartbeat; for such meddling is very surely a source of vexation, and of no other than a technical interest. Be at this hour only the gentle ministering angel sent but to soothe and comfort him who looks across the river from the borderland. Many little things you, as a nurse, will find to do; there is no need to be idle, but choose only those which help to lighten any little passing trouble—such, for instance, as moistening the poor parched lips and tongue, wiping the perspiration from the brow and face, or the changing of heated pillows for cooler ones; a gentle touch here and there is all that you will find to do, and it can be done without

A PLEA FOR THE DYING—*continued.*

haste or fuss, and always in that simple kindly way which neither asks nor looks for thanks.

Then, if one of the clergy be present, try to make the way easy for him. Do not give him the impression which some nurses do, I fear, that he is not wanted, that by his presence you are in some measure handicapped in your work. Remember it is not at all easy for him to give the consolation he would. He sometimes finds his task to be painful, though, since it is a direct duty, he has no thought of retreat. You should not be the one to render his office still more difficult. Always look upon and treat him as a friend, whatever may be his creed; for, you may be sure this will be reckoned unto you for righteousness, leavened with the God-like gift of charity. You have it within your power to do so much in this way. Do not undervalue it.

There is yet one other matter I should like to mention. It is not usual, yet it sometimes happens, that the patient may ask the nurse to inform him when the time for recovery has passed, and when it may be reasonably supposed that the hour of his death has arrived. It is a question rationally and unemotionally asked, and he has a right to expect a fair answer, but as a rule the nurse should not be the one to give it. There may, however, be exceptions. The best and the simplest reply to be given is that the doctor alone should impart this to him at the time he deems fit. In all cases you should refer to the medical man in attendance; to act on your own responsibility would be an unpardonable breach of discipline. The very rare exceptions I have remarked are those in which, from entirely social reasons, the communication would be received with less emotion from the nurse than from the doctor, who may be almost a stranger to the patient, but the information must always come from him in the first place. I cannot think we can do harm by telling the patient if we are sure his desire to know is a perfectly rational one, and not tainted with the touch of morbid fear. In the latter case we should do well to be silent; but, where the stricken one is desirous only of putting his house in order, it would be unjust to keep the truth from him.

Not long ago, a great and good man I was nursing (the head of a mighty Church) exacted a promise from me that I would not fail to tell him if I could possibly know when the last important hour dawned. I told him of the stern unwritten rule prevailing, and he was content that in his own case it should hold, only that I myself should tell him. In the end, however, there was no need to break the news to him. Instead, he it was who told me that the hours were growing short, and begged of me to plead with the doctor that all stimulants and drugs might be stopped. "Let me keep my mind perfectly clear, so that I may think of nothing else on earth, but prepare myself to go to Him who died for me," he said calmly and without any sort of emotion. His wish was carried out with the exception that I gave the injections of strychnine as before. That same evening he died with a prayer on his lips and with a great peace in his heart.

### Nursing in a German Provincial Hospital.

BY AN ENGLISH NURSE.

We English nurses would probably think ourselves overworked if we had to do what a great many German nurses are expected to do. At one hospital we went over the nurses are trained for three years and then sent out private nursing for the rest of their working days. We suggested to the nurse who was our guide that a nurse might sometime want to give up the profession for some reason; might she do so, or was she bound by any kind of religious vow? She smiled as she told us it was only the women who meant to make nursing their life-work who started on the career at all, but that they were bound by no vow. We asked what kind of salaries they received, and were surprised to learn that they received no money at all either in hospital or while private nursing. They had board, lodging, and uniform provided for them, also a home when ill or past work.

In answer to our inquiry as to times off duty, our guide said they had "no regular times, except to go to church." In the matter of annual holiday, however, we found they were more like ourselves—each having one month.

Apparently the nurses were all drawn from the peasant class, their training being very different from our own. They have no lectures nor examinations, nor do they have the same kind of training in the wards. For instance, one probationer we spoke to had just been helping at operations, and we were very curious to know how much help she could have been when we heard she had only worked in the kitchen before, having been sent straight up that morning! We asked the sister how the nurses learned the instruments and other details; she replied that they just had to pick up what they could as they went along. Fortunately German surgeons do not expect what English surgeons require. Sterilising arrangements were good and up to date, except that the sterilising drums were very small, and we found that they did not sterilise so many of the things used in the theatre as we do.

The sisters are appointed from the nurses trained in the hospital, and in most of the wards they have to work very hard. In a men's medical ward containing, I think, twenty-five beds, the doctor—who very kindly took us all over that hospital and gave us letters of introduction to two others—said there was a sister and a male attendant. Just before we were there, he told us, there had been an outbreak of enteric fever in one or two houses in the town—(a most unusual occurrence in that neighbourhood)—and the patients brought into this ward had to have cold baths constantly administered; so we could quite realise the sister must have been very busy, as she alone was allowed to superintend them.

The feeding of enteric patients we found to be practically the same as in England; but the convalescence was so much shorter—owing, I suppose, to the cold bath treatment. In passing, one detail that interested us was the little padded hammer the doctor used when percussing a chest.

Everywhere there was spotless cleanliness, all the rough cleaning being done by women employed for the purpose, and the rest of it by the probationers. There were no ward-maids.

As to hours on duty, the doctor frankly owned that he thought the nurses were overworked, and he hoped that an improvement would be made in this direction some day. The nurse told us that on alternate days they had to be on duty for thirty-six hours, but that, if work allowed, they might take a nap or two in the night. Their dining-room was a very pleasant room, with a sitting-room opening from it, containing comfortable chairs and sofas, which looked to us too little used. The nurses all dine together, except one or two told off to look after the patients, who come down immediately after the rest.

The probationers work in the kitchen and laundry when they first come to hospital, and I think I am right in saying that they do not wear caps until they go up to the wards or theatre. In the kitchen much labour is saved by electricity—all chopping, peeling, and grinding being done by pressing a button in the wall, which sets one or more of the machines to work as needed. All food is steamed.

In the laundry all was up to date, and sheets could be washed and mangled with amazing rapidity. The room looked very cheery with plenty of pretty plants in the window of the ironing-room, where heating was done by electricity.

The linen-room was in excellent order, from the scrupulously neat stacks of new sheets in the cupboards to the work waiting to be machined—and what delightful machinery, too! There was another little inviting button in the wall which, when pressed, would set in motion one or more of the six sewing-machines, and the sister showed us in a few moments how one machine would darn a hole so neatly that the darn could scarcely be seen.

It was all very interesting, but we agreed that we would rather have our own English training, although we enjoyed such luxuries as machines to darn with.

### The Impossible Pro. and Another.

One dull afternoon in November long days ago I met the Impossible probationer. Our acquaintanceship did not exceed a couple of days, yet I shall never, never forget her.

Perhaps it was her beauty which so impressed me; she was decidedly beautiful of the classical type, graceful, too. The sort of girl, I thought, as she sailed into the ward, to whom Eric Mackay would have sung his exquisite sonnets. In some way, too, she seemed undoubtedly clever, but oh! it really seems possible to be clever and yet have no common sense.

The afternoon she arrived we were very busy, several of the staff being off duty with influenza. Accordingly Nurse B. and I were sharing the duties and honours of the children's ward when Matron herself brought up the new probationer.

"Now," Matron said joyfully, "here is someone whom I hope will lighten your burdens, nurse."

The usual formalities over, I explained we were extra heavy, and would be glad if nurse would start work at once.

"We are just going to wash the children, will you wash this little fellow, his left leg is in a splint, but otherwise he is pretty well, and can show you where to find the things in his locker."

"Oh, don't ask me to wash a child," was the answer. "I've never washed one before."

"But you must make a start some day, nurse," I objected; "he isn't ill, you can't possibly hurt him."

Accordingly, in the space of half-an-hour, and after many directions from "Freddy" and some polite but timid hints as to the water being "very much hotted," and "Nurse B. don't never go to put the soap in mine eyes, she don't," the probationer's first task was finished. Then I asked her to clear away the toys which had been left out over long. After expressing her surprise at the request, she proceeded to do so, stopping to look at most of the books and winding up some of the mechanical engines. Nurse B. and I exchanged amused glances; but it was really simpler to leave her than to bother, and, after all, it diverted the big children, and so kept them good.

Presently she came across a little musical box, which highly delighted her, and this she continued to play until I suggested she should wash up the supper things, and that occupied her until it was time to go off duty.

The following morning dawned as only an English November knows how to do—misty, raw, depressing. Perhaps that is why I had no appetite for breakfast; then I was fresh at staff duty, and probably over-estimated the responsibilities. In any case the world seemed very grey as I wended my way to the ward, until I heard some hurrying unprofessional scampering footsteps behind me, and a laughing voice saying,

"Why so glum, oh staff?"

"A cloud on the horizon."

"The size of a man's hand?"

"Slightly larger; about the size of the new pro."

"Oh! fie, faint heart," divesting me of my clean apron and hanging a somewhat heavier burden on my arm, "there's no horizon to be seen. Cheer up, we'll send the pro. safely off this morning while the lions go round and roar, and this afternoon she can play with the toys while you chase clouds on the horizon and I look after the babes."

Thus often does Nurse B.—the other pro.—otherwise the "gay little Pompadour," with her cheery philosophy, smooth out wrinkles and help "roll this world a little nearer heaven." Dear little Pompadour, never shall I forget you either."

> "Just a thing of puffs and patches,
> Made for madrigals and catches,
> Not for heart-wounds, but for scratches."

Having arrived at the ward and exchanged greetings, before doing our own tasks we set the new probationer the simpler ones, most of which, by the way, we did over again when she had gone at 10.30.

Then came the lions, to quote Nurse B.; their roaring was mild and gentle; altogether the morning's work was quite a success.

At 2 P.M., chiding myself for glum forebodings, I sent Nurse B. off duty, thinking the probationer and I would manage.

I often wonder if accidents *are* accidents, for they so consistently come when least desired that they seem rather like some well-thought-out plan to further our education in the way of discipline. However that may be, nurse had just been away an hour when the house-surgeon came up to say he had admitted a child, and wanted to perform a slight operation immediately.

The children's ward is divided into two, with separate doors reached by a small narrow passage, so I ran across and told nurse I should be engaged for a quarter of an hour or so. Would she refill all the hot bottles and cut up the bread and butter for tea? "If you should want anything particularly come and ask me," I said—which she did.

The assistant house-surgeon had just got the patient under and the surgeon had taken up a scalpel, when the door was thrown wide open and in glided the probationer, wreathed in smiles. Having glanced from the two men to myself, as one who expected an introduction, she asked where the musical box was. "In the cupboard with the toys, nurse." "Oh, thanks, all right"—and she departed. The house-surgeon looked up and we both laughed.

"Don't look so tragic, nurse; is she a new hand?"

"Yes, Mr. Dawson."

"Rather hard lines just now, but still half a loaf's better than no bread, eh!"

"Oh, most decidedly," said the susceptible assistant, whose eyes had sorrowfully followed the probationer's exit.

The operation was soon completed, and, the after effects of the anæsthetic over, I ran across to see how nurse was getting on.

I went to the kitchen first, intending to put on the milk for tea. There was a pretty kettle of fish. The kettle stood in the sink, lid on floor, tap running, and the stove cheerfully burning in mid-air.

I re-arranged these details and proceeded to the ward. My

THE IMPOSSIBLE PRO. AND ANOTHER—*continued.*

first impression there was that the probationer had certainly filled the hot bottles, not wisely, however, but too well. She had put them in at the foot of each cot, but not tucked in the bed-clothes again. One bottle had been left on the centre of the cot, while the occupier sat on his pillow to avoid contact with it, glaring meanwhile at the unconscious pro. in sullen defiance.

Later I discovered yet another, on which the top had not been screwed sufficiently tight. And oh! well for her, as for me, that having mislaid the bottle-bag she had put it in a foot blanket, through which, fortunately, the water had not yet penetrated. Yet she had some idea of warmth as a necessary agent in the gentle art of healing. The fire, overladen with lumps of coal, was roaring up the chimney in a manner calculated to act as a powerful bleacher to every member's head on the "Weekly Board," could they but have seen it.

There, on the rug, a sublime spot in the midst of what would shortly be a "howling wilderness," with a case of chorea perched opposite to her, sat the "impossible pro." playing the musical box with a happy air of gay abandon and irresponsibility.

Alas! for human nature, although my own salad days were still green and crisp in my memory, tears, not of "divine despair," but evil passion, came to my eyes; if the royal "wonderland" guests played croquet after this manner, no wonder at all the queen of hearts cried, "Off with their heads," I thought.

To gain time I removed some of the superfluous lumps of coal; then I turned and surveyed once more the picture on the rug. Very pretty it was, too, though I didn't think so at the time.

"Nurse," I remonstrated, "just look what a frightful state you have made the ward in, and really you must know we are here to work, and not to amuse ourselves."

"But," indignantly, "I've done all you asked me to do, and now I'm keeping this little thing with jerks quiet."

Thinking there'd soon be a big thing with jerks to keep quiet, and wondering if any "first-aid" hand-book gave directions for the treatment of impossible pros., I picked up the hot, excited little patient and tucked her safely in bed.

The probationer's eyes followed me in resentful indignation as a spoiler of sport. Then she slowly rose, stretched her arms, and asked, "What shall I do now?"

I glanced at the clock, then, "Suppose you go and get some tea, nurse; it's nearly four o'clock, and, oh! well, perhaps you're tired, don't hurry back."

"Oh, all right"—and she didn't.

Then I proceeded to straighten trifles. It has been said that if Cleopatra's nose had been a different shape the world's destiny might have been changed. I can well believe it.

Nurse B. tripped up at 4 and found me rescuing the prize baby's toes from the close proximity of a fast-oozing bottle and damp blanket.

From her point of view it was apparently funny. Ah, well! hasn't some sage accounted for that by saying, "Each man sees around him what each man brings the faculty of seeing," or words to that effect? An old-fashioned sage, but I suppose he would allow woman some faculty for seeing also.

"Forgive me, nurse," the Pompadour said presently, mopping her eyes, "but you do look funny when you're savage."

The Pompadour respects no one save her coiffeur, and I said so. She ignored the remark.

"Oh, dear! you won't get off again to-day," she said.

"I shall to-morrw," I growled, and I did.

The sentry found me at 5.30 A.M. with a touzzled head and temperature 103.

"Got it too? What a bother!"

Thus I turned my face to the wall and "remembered happier days."

But not for long; later in the day, after an imperative tat, tat, tat, in came the gay little Pompadour like a sunny breeze, scattering nerves and reflections like so much dust.

"I knew it was you," I said gaily, feeling happily frivolous all at once, "I heard your butterfly wings on the staircase."

The Pompadour's dancing eyes suddenly took on a look of alarm, and wandered sorrowfully from medicine bottle to chart, and back to my face again.

"Mr. Dawson didn't mention anything about temporary insanity," she rudely murmured, sotto voce, "he said it was a simple case of flu."

"So it is."

"Tut, tut, weak diagnosis and total lack of observation; you'd never get a tempo on so slight a provocation. Ah me! what a pity you didn't let fly yesterday—murder will out—and now what shall we do without you?"

The sudden change in her voice from banter to genuine regret came as a revelation, and gave cause for a hurried reply.

"The babes will certainly all die sudden deaths, and the jury bring in a verdict of heart failure caused by the absence of the staff nurse during a period of temporary insanity. Lower the blinds please, my eyes ache."

And I thought as I watched her what a grey place the world would be without a gay little Pompadour or so.

### An Afternoon in a County Gaol.

BY A NURSE.

I had been asked on one or two occasions to give a "talk" to the female prisoners in a neighbouring gaol, and though my first impulse was to say "I couldn't possibly," I was induced to take the place of a friend who was prevented from going at the last minute. The lady visitor who had invited and was responsible for me (a member of the Prisoners' Aid Society, I believe) met me at the station and took me to the gaol, which presented a formidable but rather picturesque appearance. The man in charge had been advised of our visit and admitted us into a small courtyard; he then unlocked another heavy iron gate and we passed through into a neat yard laid out with grass. On arriving at yet another gate a wardress answered the ring, and we found ourselves in the women's side. The building rose like a grim, dull-looking hospital, only the large, bright-looking windows with occasional glimpses of beds within, were missing. Surrounding this building were asphalt paths with well-kept grass. This forms the exercise-yard. Here, one of the wardresses told me, the women have to walk at a certain distance from each other, and many are the stratagems adopted for getting near enough to speak to one another, a favourite one being to stop and tie up their shoelaces.

We were received by the head wardress in her office. She was a bright, cheery woman, and her conversation enlightened me a good deal about some of the work done by the Prisoners' Aid Society. The members visit frequently and so become acquainted with the prisoners, and offer much kindly advice and help.

The corridors were very light and not nearly so gloomy as I expected to find them. Perhaps one thing was that just then the doors of the "well-behaved" were standing open, as was the rule for a stated time in the afternoons. An iron staircase ran up to the top in the centre, so that much could be seen and heard from the ground floor. About thirty or forty women were waiting for us in the infirmary ward, a bright room with a cheerful fire, four or five beds, and a corresponding number of windows. Each had carried her own stool from her cell, and it struck me that they certainly looked very stolid and depressing. I thought it would be very hard work to interest them, because what could be the use of talking about fresh air or how to nurse their children when they were shut up there? But soon I was surprised at the animation they showed, quite ready to laugh at any opportunity given, and when a wardress (there were two or three in the room) asked for one to act as a sham patient when I was bed-making there was great excitement and much merriment. Many were intelligent and evidently took in something of what I said, for on my second visit one of them was most anxious to tell me that my remedy of holding the head down for an attack of slight faintness had not answered in her case, as she had felt very faint one night and had got out of bed many times to put her head down, but with no result. Poor woman, she could get no more air and it happened that the cells were all very warm that night!

At the close they were invited to ask me any questions they liked. One inquired "what to do when the baby had red eyes after measles?" and another "what to do when the baby had a fit?" However, my pleasure at their interest was rather spoilt when I heard that both those women were there for neglect of their children! A nurse who was unhappily there (she was not a fully-trained nurse I was glad to hear) did not wish to leave her cell, and I gathered that she had been gradually getting lower and lower for some time past, and had finally been found collecting money for an imaginary patient.

The majority were there for drunkenness and immorality, extremely few for their first time. Many had made over fifty appearances, and some even over a hundred. They wore a kind of Sister Dora cap with a tape under their chins, which apparently kept the caps on their heads, though even then they certainly had a difficulty in keeping them straight. They also wore dark blue washing dresses, holland aprons, and on the left side of their chest a round red tab, rather bigger than the palm of one's hand, with a number on. The wardresses seemed such nice kindly women, possibly about thirty years of age, and dressed in navy serge, and they also wore, indoors as well as out, little dark blue bonnets, like nurses. When I had finished talking the women all took up their stools and filed out, and it was only then I discovered that we had been locked in. I suppose the doors lock when closed. The head wardress kindly showed me through some of the building. The cells were a step lower than the corridor, and lighted by a window high in the wall, but a good sized one, and though the floor was of stone, and it was winter time, it did not strike me as cold. The bed, I think, was a spring with a flock mattress. The metal plates and mugs, all well polished, stood up against the wall, and above these was a little bookshelf, containing a Bible, hymn-book, and in cases of Roman Catholics one or two other little books. After a month the prisoners are allowed a book from the library at stated times. The padded cell was far more comfortable than any I had seen, the walls and floor being covered with a very deep kind of cocoa fibre, much thicker than cocoa-matting; it was so much more cosy and warm-looking than the ordinary padded cell. The nurses' place for supervision was above. I was surprised to hear that they had very few cases in the infirmary ward. Two babies born in prison were shown to me, very bonny, bright little things they were and apparently quite as much spoilt as are our hospital babies.

### Rural Nursing.

BY A NURSE IN THE NORTH.

For two years I belonged to the staff of a Rural Nursing Association in one of our northern counties, and my experience, I fancy, may prove interesting to those who have never worked in country districts. A great deal of maternity nursing came to my share. I had a midwifery certificate and I had had some general training, though not three years'. This training, however, I found most useful. My work lay over a large area of country, and was principally in the villages and outlying farms, although sometimes we had cases in towns, and occasionally did duty in hospitals. I should say that the nurses who formed the main body of the Association served for four years, during two of which they were training, and the remainder doing private work; but I belonged to a few who were taken on a special agreement. As for our life between cases, the home was a real home to us, and very merry we were amongst ourselves; I am sure many of us often look back regretfully from our various posts to those pleasant days. The Association was primarily intended for the sick poor, so the greater percentage of the cases were cottage ones, and the maternity ones were very hard, as the nurse had to do "all for the comfort of the family except the washing," and it is no light task to cook and keep clean for a family as well as look after a woman and a baby; but I met with a lot of gratitude from the simple countryfolk, and the husbands in most cases rose to the emergency and lighted the fire and carried the water, and on Sundays helped with the cooking, to assist "nurse." Then the children—what amusing little creatures they were! One little boy of two years was an especial character. His father was a country postman, and at night Joe would listen attentively for the first sound of wheels to announce the return of "mine father." His wishes were always prefaced with "Boy wants," and Boy often wanted.

Many of my cases lay in the dale country, and I do not believe there can be a prettier part of England than that land of hills and waterfalls. In one house I was almost close to a fall, and as it was winter time the noise of the water falling over the rocks kept me awake at nights till I got used to it.

Off-duty time was necessarily erratic, but when there was a kind neighbour or a big daughter it was easy to get for a ramble in an afternoon, and often other nurses would be in the same neighbourhood and exchange visits. Of course, in the summer time there were the evenings when the husband returned from his work and could be left in charge.

My most unpleasant case occurred amongst a higher grade than the cottage ones. The people were farmers, and the farm an outlying one on a hillside. In addition to this it was the month of January and snow had fallen, so it can be imagined how isolated my position was. My patient had had terrible hæmorrhage and been put to bed in the kitchen, and during the six weeks I was with her was only lifted out to have her bed made. During this time the husband returned home intoxicated some three nights in the week, and had to be humoured and persuaded to go upstairs and leave me in possession of the sick-room. I have learnt since that I gained a reputation in the neighbourhood for being brave and good, but I know I was often quaking inwardly at the thought of possible complications.

My longest case was one of head injury to a youth.

I do not specify the nature, for during the ten weeks I was with him no definite name was given to the complaint. During all that time the lad lay unconscious or occasionally semi-conscious, but although an operation was discussed it was never resorted to. My astonishment was great when, some three months later, he came to see me. I did not know him when I opened the door to him, so greatly had he altered; but, strangely enough, he knew me. He said he had no idea of what I should be like, but directly he saw me he felt that he had known me before, and recognised a cameo brooch I always wear.

I suppose I must call a placenta-prævia my most interesting case, and, indeed, the woman made a marvellous recovery. This was one of my cases in the land of hills and waterfalls, and it was lovely to watch the sun rise over the hills in the early summer mornings, for here I was on night duty at first, and had a big daughter to do most of the housework.

Amongst other accomplishments during my rural nursing I learnt to bake, and will warrant my pastry to compete with that of a professed cook; and if our work could not be called altogether nursing, it was evidently very much wanted, for many of the women required skilled attendance and yet could not afford to have all their work done exclusive of the nurse. Of course, there are many awkward circumstances for a nurse, chief of which are the sleeping arrangements. Although in most cases I had a separate bed, a separate room was a rare luxury. I always took my meals with the family, and notwithstanding that the fare was simple I do not think my health ever suffered from it. Looking back I can say that I met with uniform kindness, and shall never regret coming so closely in touch with my suffering sisters of the working class.

### Everybody's Opinion.

[Correspondence on all subjects is invited, but we cannot in any way be responsible for the opinions expressed by our correspondents. No communication can be entertained if the name and address of the correspondent are not given as a guarantee of good faith, but not necessarily for publication. All correspondents should write on one side of the paper only.]

### THE MATRONSHIP OF DURHAM COUNTY ASYLUM.

"Mental and Hospital Trained" writes: Your "note" *re* the matronship of the Durham County Asylum was not, I hope, your own sentiments, but was prompted by the information of a petty impotent person. The law is the place to resort to if wrongfully dismissed. Speaking of the popular little lady who is now matron, allow me to remind you that if promotions were fair in our asylums she should have held the present post years ago. Miss Jones's election to the post of matron is a triumph for all asylum nurses; too long have they worked under the grave disadvantage of having no chance of promotion to the higher posts in their profession, these usually being filled by hospital trained nurses who shrink with horror and disgust at actual asylum nursing. I regret to say medical superintendents are our greatest enemies to promotion; but a rule that acts harmoniously on the male side of an asylum should be equally applicable to the female side. The new matron will be hailed with delight by all whose good-will is worth having, and I feel sure that the dear old Durham County Asylum will return to the good times which were enjoyed previous to the advent of the late matron.

[Our correspondent is mistaken; we expressed no sentiments whatever about the appointment.—Ed. The Hospital.]

### THE NURSING QUESTION IN PARIS.

"An English Nurse" writes: I have read M. Paul Lutard's article with great interest. He deplores the fact that in France the daughters of professional men will not embrace the nursing profession. May I suggest what seems to be a very powerful reason against their doing so? I refer to the want of distinction which, from his article, appears to exist between the professional or nursing staff and the domestic servants. M. Paul Lutard says that a probationer who wishes to enter the hospital serves for a year, and then the department places her either among the class of *personnel soignant,* if she has had instruction or possesses a diploma from some nursing school, or if not, among the class of *personnel servant* or domestic servants. This statement is difficult for an English nurse to understand, as it refers to entirely different conditions to those obtaining in the nursing profession in England. For, of course, with us every probationer "has instruction"; it is for that very purpose that she goes to a hospital, and the hospital itself is the "nursing school"; no "nursing schools" apart from hospitals and infirmaries exist. Also they only grant diplomas or certificates to their pupils on the completion of their training. With us, of course, a probationer enters a hospital for the purpose of being trained as a nurse, and the domestic servants, whether kitchenmaids, housemaids, laundresses, porters, etc., apply for situations at hospitals in those capacities just as they would at hotels, clubs, or private houses. The only servant specially belonging to a hospital, the wardmaid, is simply the domestic servant who does the cleaning and housework of a ward, as the kitchenmaid and housemaid do in their departments, and has no more to do with the nursing than they have. I think that, generally speaking, it would be equally impossible to induce the daughters of doctors, merchants, professors, etc., in England to become nurses if they were obliged to enter hospitals for training where they and the porters, wardmaids, kitchenmaids, scrubbers, etc., were all probationers together, and where the would-be nurse could be placed on the nursing staff or turned into a kitchenmaid at the discretion of the authorities governing the hospital, whether a Government department, as in France, or a hospital board consisting of medical men and lay subscribers, as with us. Such a condition of things would make a hospital training almost impossible to girls of gentle birth, whether in England or in France.

### Appointments.

[No charge is made for announcements under this head, and we are always glad to receive and publish appointments. The information, to insure accuracy, should be sent from the nurses themselves, and we cannot undertake to correct official announcements which may happen to be inaccurate. It is essential that in all cases the school of training should be given.]

Devonport Workhouse Infirmary.—Miss Madeline Jane Bevan has been appointed charge nurse. She was trained at Paddington Infirmary, and has since been district nurse and midwife at Stroud.

East Sussex Hospital, Hastings.—Miss Winifred Beckett has been appointed staff nurse. She was trained at Huddersfield Infirmary, where she took temporary duty as charge nurse, theatre sister, and night superintendent.

Gravesend Hospital.—Miss Annie M. Ford has been appointed sister. She was trained at the Devon and Exeter Hospital, and has since been staff nurse at the Royal Bath Hospital, Harrogate, and night sister at Windsor Royal Infirmary.

Jenny Lind Infirmary for Children, Norwich.—Miss Elizabeth Esbelby has been appointed sister. She was trained at the Children's Hospital, Gateshead, and the General Infirmary, Chester, and has since been ward sister and night sister at the Surgical Hospital, Stockton-on-Tees.

KNIGHTSWOOD HOSPITAL, GLASGOW.—Miss Ella M. Patisson has been appointed charge nurse. She was trained at the City Hospital, Edinburgh, and the Western Infirmary, Glasgow.

LEEDS SANATORIUM, GATEFORTH, SELBY.—Miss Emily E. Jackson has been appointed matron. She was trained at the Royal Infirmary, Bradford, where she was afterwards sister and housekeeper. She has since been assistant matron and home sister at the Union Infirmary, Fir Vale, Sheffield.

NOBLE HOSPITAL, DOUGLAS, ISLE OF MAN.—Miss Esther Bridson has been appointed matron. She was trained at the Royal Southern Hospital, Liverpool, where she has since been sister.

QUEEN VICTORIA JUBILEE INSTITUTE FOR NURSES.—Miss A. C. Halliday has been appointed inspector. She was trained at the Sussex County Hospital, and was appointed Queen's nurse January 1, 1900. She has held the post of superintendent of the Hampshire Nursing Association since May 1902.

ROYAL INFIRMARY, NEWCASTLE-UPON-TYNE.—Miss Lydia Harvey Rigbey has been appointed night superintendent. She was trained at St. Bartholomew's Hospital, London, where she was subsequently staff nurse and sister. She has since been sister at the Samaritan Free Hospital, London.

SANDBACH WORKHOUSE HOSPITAL.—Miss Elizabeth Roberts has been appointed superintendent nurse. She was trained at Birmingham Infirmary, and has been nurse at Spalding Union Workhouse and Leek Union Workhouse.

SHERBORNE UNION, DORSET.—Miss Alice Dunford has been appointed charge nurse. She was trained at the Ecclesall Infirmary, Sheffield. She holds the certificate of the Central Midwives Board.

SOUTHALL SANATORIUM.—Miss Jessie E. Cooke has been appointed nurse-matron. She was trained at Chorlton Poor-law Infirmary, and has since been charge nurse at one of the fever hospitals under the Metropolitan Asylums Board, ward sister at Ruchill Fever Hospital, Glasgow, sister at Coventry Fever Hospital, and sister at the Central Sick Asylum, Hendon.

WOLVERHAMPTON AND STAFFORDSHIRE GENERAL HOSPITAL. Miss Henrietta Hannath has been appointed superintendent of nurses. She was trained at King's College Hospital, London, and has since been assistant matron at the General Hospital, Birmingham.

### To Nurses.

WE invite contributions from any of our readers, and shall be glad to pay for "Notes on News from the Nursing World," "Incidents in a Nurse's Life," or for articles describing nursing experiences at home or abroad dealing with any nursing question from an original point of view, according to length. The minimum payment is 5s. Contributions on topical subjects are specially welcome. Notices of appointments, letters, entertainments, presentations, and deaths are not paid for, but we are always glad to receive them. All rejected manuscripts are returned in due course, and all payments for manuscripts used are made as early as possible after the beginning of each quarter.

### Notes and Queries.

**REGULATIONS.**

**The Editor is always willing to answer in this column, without any fee, all reasonable questions, as soon as possible.**

**But the following rules must be carefully observed.**

1. **Every communication must be accompanied by the name and address of the writer.**
2. **The question must always bear upon nursing, directly or indirectly.**

**If an answer is required by letter a fee of half-a-crown must be enclosed with the note containing the inquiry.**

*Study*

(111) Is there any way in which I could improve my position? I hold the L.O.S. and C.M.B. certificates, but for family reasons I am unable to leave home for some time. I hope to get a resident patient, and until I do I should like to study for something, to improve my standing later. Could I do any good at gynæcology? Massage would be out of the question in the country.—*A. E. L.*

Certainly gynæcology offers a wide scope for study and interest. A really thorough knowledge of it is invaluable to any nurse, especially one holding your certificates. Much can be learnt theoretically of the preparation of, attendance at, and after-nursing of special operation cases.

*Ownership of Uniform.*

(112) In a certain institution the nurses are given material for their dresses; the expense of making, lining, and buttons has to be paid by the nurse. If a nurse leaves at the end of a year are the dresses hers, or do they belong to the institution? If to the institution, should the committee pay back the expense of making, etc.?—*Chris.*

The uniform would certainly belong to the institution, and the nurse must pay for the making of the dress, etc. She must remember that she has had value for the outlay in the wear of the garment.

*Invalid Bed Table.*

(113) Can you inform me if there is any place where an invalid bed table could be bought second-hand, or hired cheaply, or if there is any society where one could be procured for a poor person?—*S. P.*

You might inquire at Bailey's, 38 Oxford Street, if they lend such articles. You do not say what kind of table you need; if it is only a simple one with four legs deep enough to allow of the table passing over the patient's body, any carpenter will make you a plain deal one for 3s. or 4s.

*Masseuses.*

(114) Is the certificate of the "Incorporated Society of Masseuses" the best one to obtain for massage? What are the best and quickest means of qualifying for and obtaining it, and the best books to study?—*L. L. D.*

Write fully to the Secretary, the Incorporated Society of Trained Masseuses, 12 Buckingham Street, Strand, W.C. "The Art of Massage," by Mrs. Creighton Hale, is a useful book to study.

Will you kindly tell me the best and cheapest way to obtain training in massage with a certificate, or is there any hospital which teaches trained nurses for a small fee? Also would a certificated masseuse find employment in London in any establishment other than a nursing home?—*A. F. G.*

See reply to *L. L. D.* The profession is much overstocked, and it is difficult to get sufficient employment to make it lucrative.

*Maternity Nurse.*

(115) Being a trained and certified monthly nurse, and just starting a connection, will you tell me the best way to get cases? Are there any associations I could belong to?—*Anxious.*

You will find it uphill work at first, and must have patience. Have your professional cards printed, and call on the doctors of the town in which you live. Then, when you have had two or three cases and given the doctors satisfaction, things will become easier. We doubt whether there is an association which can help you.

*To Study Maternity Work.*

(116) I am thinking of taking up maternity work. Kindly let me know which is the best way to commence.—*L. W.*

You can receive training at any of the lying-in hospitals. Write to the various matrons for particulars.

*London County Council.*

(117) The London County Council are giving a course of nursing lectures. Can you tell me where I can obtain particulars concerning them?—*A. E. G.*

Write for particulars to the Clerk to the London County Council, Spring Gardens, S.W.

*Probationer of 19.*

(118) Can you advise me where to train in a children's hospital. I am 19, and have been in the nursery five years. I have also had a course of home nursing given by the L.C.C. evening classes.—*L. P.*

No hospital will receive probationers of 19 years of age. Wait until you are 21, and then consult "How to Become a Nurse," published by the Scientific Press, 28 Southampton Street, Strand.

*Nurse-Matrons.*

(119) Will you kindly tell me if appointments for nurse-matrons of small fever hospitals are made through the medium of agencies? I have answered several advertisements which have appeared in The Hospital for such posts, and have very seldom had even an acknowledgment of my application.—*Scott.*

Small fever hospitals generally advertise when such an appointment as you mention is vacant, unless offered to former nurses. But applications are so numerous that it is possible only a few selected ones are answered. You cannot do better than watch our advertisement columns.

*Daily Work.*

(120) (1) Is it possible for a trained nurse (three years' certificate) to obtain daily work in or near London? I am anxious to live at home and get daily work if possible. (2) Do the London County Council require many nurses in their schools, for invalid children?—*Stockwell.*

(1) Write for particulars to the Marylebone Association, 126 Seymour Place, Marylebone, N.W. (2) Write to the London County Council, Spring Gardens, S.W.

*Home for Aged Relative.*

(121) Is there a home or institute where I could place an aged relative (82)? She is in very good health, but cannot express herself clearly. The doctor says she is suffering from senile decay. She is without means, but I should be quite willing to pay from 5s. to 8s. per week towards her support.—*F. F. H.*

You might write to the Hon. Secretaries, Homes for the Aged Poor, 86 Penge Road, Anerley, S.E., or to the Aged Poor Society, 48 Rockford Street, Lismore circus, N.W.

*Massage.*

(122) I wish to learn massage. Will you kindly tell me where I could gain the most satisfactory result?—*Miss S. F.*

Write to the Secretary, the Incorporated Society of Trained Masseuses, 12 Buckingham Street, Strand, W.C. Or refer to our advertisement columns.

*Midwifery Bag.*

(123) Can you tell me where to get a second-hand midwifery bag fitted for a poor woman who has just got her certificate at the City of London Lying-in Hospital, and is not able to afford to buy a new bag?—*Mrs. F.*

You might obtain one by advertising in The Hospital.

*Training.*

(124) Please kindly tell me (1) if there is any hospital where I could enter before the age of 21. (2) Also, do you know of any "first aid" classes in Bayswater which I could join?—*O. C.*

(1) A few children's, provincial, and cottage hospitals receive probationers at 20 years of age. Study "How to become a Nurse." (2) Write to the Secretary, St. John's Ambulance Association, St. John's Gate, Clerkenwell, E.C.

*Moving Helpless Patient.*

(125) I should be glad if you could tell me through your columns the best way for me to move a patient who has had a stroke and is quite helpless. She came to some friends on a visit, and was taken ill the next day, and we are anxious to get her home. She will have to go a short railway journey.—*Anxious.*

The simplest way would be to convey the patient on a stretcher. If you cannot hire one privately, a hospital or ambulance association would doubtless lend one. The district nurse or members of the ambulance would give you help in lifting the patient into the train. Make arrangements with the railway authorities for the use of a reserved compartment.

**Handbooks for Nurses.**

| | Post Free. |
|---|---|
| "How to Become a Nurse: How and Where to Train." | 2s. 4d. |
| "Nursing: its Theory and Practice." (Lewis.) ... | 3s. 6d. |
| "Nurses' Pronouncing Dictionary of Medical Terms." | 2s. 6d. |
| "Complete Handbook of Midwifery." (Watson.) ... | 6s. 4d. |
| "Preparation for Operation in Private Houses." ... | 0s. 6d. |

Of all booksellers or of the Scientific Press, Limited, 28 & 29 Southampton Street, Strand, London, W.C.

### For Reading to the Sick.

### IN SEARCH OF REST.

There's peace and rest in Paradise,
In weary hours we say;
And oh that we had wings like doves
That we might flee away!

At times, through the long lonely watch,
Nor sun nor moon appears;
Without, incessant fightings are,
Within, incessant fears.

Then for the quiet land we long,
And the abode of Peace;
And for the word "Come, weary soul,
From war and vigil cease!"

*J. R. Vernon.*

The Psalmist says, "Oh that I had wings like a dove! for then would I flee away, and be at rest." He wrote this when he was in great trouble. And sometimes when we are in trouble, and tired, in this disappointing world, we, perhaps, wish that we had the wings of a dove that we might flee away, and be at rest. But we have the wings of the Dove, the Holy Ghost; and in meditation we can spread those wings, and flee away from the things of earth to heaven itself, and rest there awhile in the contemplation of divine truth, and in communion with our dear Lord!

But you may ask, "How can I be sure of persevering to the end?" The grace of perseverance, like that of regeneration, is a grace which we cannot merit, but which God gives to those who seek it earnestly in prayer. The great help, however, to perseverance, next to prayer, is, never to give up any spiritual exercise which we have begun, unless, indeed, we are quite sure that we are giving it up for a good reason. Do not, in your rule of life, undertake too much, and so have to give up certain things, because they are beyond your strength.

The remedy is, to be earnest and collected at the time of prayer, and to use prayer so perseveringly, that it may develop in us the virtue of Hope, and that Hope will be the spring and stimulus to an active and happy spiritual life.

There must be in our spiritual life seasons of darkness, coldness, and desolation; there must be night and day, summer and winter, just as there are in our physical life. But at such times we must exercise the virtue of faith and trust in God, and fall back upon our remembrance of God's goodness to us in the past.

*Rev. J. G. Mortimer, D.D.*

We fain would tread the famous way
Martyrs and saints have trod;
The hours ebb fast of this one day
Of noblest war for God.

The Lord Himself hath need of us
Until the fight is won;
And the King's words shall thrill the heart:
"Servants of God, well done!"

*J. R. Vernon.*

